# The Adenosine A2A Receptor Activation in Nucleus Accumbens Suppress Cue-Induced Reinstatement of Propofol Self-administration in Rats

**DOI:** 10.1007/s11064-021-03238-9

**Published:** 2021-02-22

**Authors:** Zhanglei Dong, Bingwu Huang, Chenchen Jiang, Jiangfan Chen, Han Lin, Qingquan Lian, Binbin Wu

**Affiliations:** 1 Department of Anesthesiology, Perioperative and Pain Medicine, The Second Affiliated Hospital and Yuying Children’s Hospital of Wenzhou Medical University, 109 Xueyuan West Road, Wenzhou, 325027 Zhejiang China; 2 Clinical Research Unit, The Second Affiliated Hospital and Yuying Children’s Hospital of Wenzhou Medical University, Wenzhou, China; 3 Molecular Neuropharmacology Laboratory, Wenzhou Medical University, Wenzhou, China

**Keywords:** A2A receptor, D2 receptor, Propofol, Cue-induced drug-seeking, Relapse

## Abstract

Propofol has shown strong addictive properties in rats and humans. Adenosine A2A receptors (A2AR) in the nucleus accumbens (NAc) modulate dopamine signal and addictive behaviors such as cocaine- and amphetamine-induced self-administration. However, whether A2AR can modulate propofol addiction remains unknown. AAV-shA2AR was intra-NAc injected 3 weeks before the propofol self-administration training to test the impacts of NAc A2AR on establishing the self-administration model with fixed ratio 1 (FR1) schedule. Thereafter, the rats were withdrawal from propofol for 14 days and tested cue-induced reinstatement of propofol seeking behavior on day 15. The propofol withdrawal rats received one of the doses of CGS21680 (A2AR agonist, 2.5–10.0 ng/site), MSX-3 (A2AR antagonist, 5.0–20.0 μg/site) or eticlopride (D2 receptor (D2R) antagonist, 0.75–3.0 μg/site) or vehicle via intra-NAc injection before relapse behavior test. The numbers of active and inactive nose-poke response were recorded. Focal knockdown A2AR by shA2AR did not affect the acquisition of propofol self-administration behavior, but enhance cue-induced reinstatement of propofol self-administration compared with the AAV-shCTRLgroup. Pharmacological activation of the A2AR by CGS21680 (≥ 5.0 ng/site) attenuated cue-induced reinstatement of propofol self-administration behavior. Similarly, pharmacological blockade of D2R by eticlopride (0.75–3.0 μg/site) attenuated propofol seeking behavior. These effects were reversed by the administration of MSX-3 (5.0–20.0 μg/site). The A2AR- and D2R-mediated effects on propofol relapse were not confounded by the learning process, and motor activity as the sucrose self-administration and locomotor activity were not affected by all the treatments. This study provides genetic and pharmacological evidence that NAc A2AR activation suppresses cue-induced propofol relapse in rats, possibly by interacting with D2R.

## Introduction

Drug use disorder results in global health issues and massive economic losses, and relapse is the severest limitation and barrier to successful drug use disorder treatment after periods of abstinence [1, 2]. Propofol, an intravenous anesthetic primarily used for anesthesia induction and sedation, has been reported as a substance for addiction. Increasing propofol addiction is reported in many countries, especially among academic anesthesiologists and nurses over the past decades [3–6]. Propofol addiction has also been successfully modeled with self-administration and condition place preference (CPP) in rats [7–9]. Humans and preclinical studies provide evidence that drug craving and relapse can be triggered by acute re-exposure to the drug, drug-associated environmental cues or stress [10–12]. Our animal study has demonstrated that propofol relapse behavior in rats can be induced by previous drug-paired cues[13].

Some brain regions are implicated in the development of drug reward, craving and relapse, and the foremost among these regions include a mesolimbic dopaminergic system that projected from the ventral tegmental area to the ventral striatum of nucleus accumbens (NAc), which is considered as the common final pathway of drug reward [14]. Multiple drugs implement their reward effects by increasing the dopamine (DA) level in NAc by promoting DA release or inhibiting DA reuptake [15, 16]. In the striatum, DA acts on dopamine D1 and D2 receptors (D2R), and activate direct (striatonigral) and indirect (striapallidonigral) projection of the medium spiny GABAergic neurons (MSNs) by stimulation of D1 receptor and D2R in NAc, respectively, and D1 receptor and D2R play different roles in mediating drug use disorder. DA facilitates the direct pathway activity through the D1 receptor but inhibiting the indirect pathway through the D2R [17]. Our previous studies suggested that the mesolimbic dopaminergic system is involved in modulating propofol self-administration mainly through the D1 receptor in the NAc [7, 8, 18], and the DA transmission in the basolateral amygdala (BLA) is indicated in propofol relapse [13]. However, whether dopaminergic transmission in the NAc is involved in propofol relapse remains to be elucidated.

DA effects on drug reward were affected by other neurotransmitters. Adenosine is a well-known modulator of DA neurotransmission and neuronal activity. Drug abuse elevated adenosine in the brain, and A2A receptor (A2AR) has been indicated a novel target for drug use disorder and relapse treatment [19]. Adenosine A2AR in the NAc was reported to play a key role in drug self-administration relapse behavior of cocaine, morphine, nicotine and ethanol [20–23]. Wydra et al. demonstrated that intra-NAc microinjections of A2AR agonist of CGS21680 dose-dependently attenuated cocaine reinstatement behavior, while evoked the cocaine reinstatement seeking behavior by the antagonist of A2AR in the NAc, these findings indicating A2AR in the NAc might be critical for drug relapse [24].

Adenosine is medicated by subtypes of adenosine receptors, including A2AR, which is highly expressed in striatum and mostly postsynaptically colocalized with D2R on striatopallidal GABA ergicneurons of the indirect pathway with an antagonistic interaction [25]. Adenosine A2AR exerts tonic inhibitory control over D2R signaling within the striatum, and the two receptors present opposite effects on physiological and molecular responses, such as motor behaviors and neurotransmitter release [26]. The A2A-D2 receptor heteromer participated in the regulation of drug use disorder. Chronic cocaine administration enhanced the D2R by reducing the expression of A2A-D2 receptor heteromer [27], and an important regulation via D1 receptor and D2R in propofol relapse has been suggested [13]. Nevertheless, whether adenosine A2AR and D2R in the NAc underlying propofol relapse is poorly understood.

In the present study, we examined the effects of the adenosine A2AR in the NAc on propofol self-administration model establishment and the cue-induced reinstatement of propofol self-administration behaviors. The effects of D2R in the NAc on propofol relapse were also examined to explore the possible underlying mechanisms. The sucrose self-administration and locomotor activities were tested to identify the specificity of A2AR and D2R on propofol relapse.

## Materials and Methods

### Animals

**Fig. 1:**
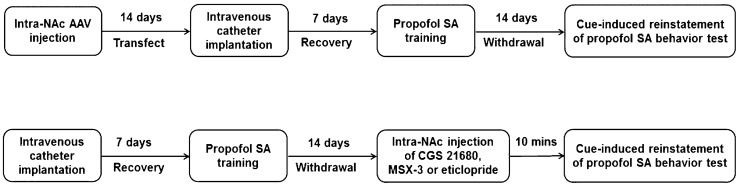
The schematic of the timeline of the rats that received stereotaxic injections in the NAc for behavior test. *SA* self-administration

Adult male Sprague–Dawley rats weighing 250–300 g (8–9 weeks old) were purchased from the Experimental Animal Center of Wenzhou Medical University, and all experimental procedures were approved by the Animal Care and Use Committee of Wenzhou Medical University. The rats were individually housed in a temperature-controlled room (22–24 °C) with a 12-h light/ dark cycle and were free to food and water. Only the rats were successfully implanted with chronic indwelling catheters via jugular vein, and guide cannulae in the NAc and completed propofol self-administration training were randomly assigned to either vehicle (control) or one of the treatment groups after a 14-day abstinence period. The schematic of the timeline of the procedures is presented in Fig. 1.

### Drugs

In the present, propofol adopted was obtained from AstraZeneca (10 mg/ml, Diprivan, Italy), which was daily prepared for training. A single dose of 1.7 mg/kg/injection was used for self-administration behavior training with the previous study results [28]. The A2AR agonist CGS21680 (4-[2-[[6-amino-9-(N-ethyl-b-D-ribofuranuronamidosyl)-9H-purin-2-yl]amino]ethyl]benzenepropanoicacid hydrochloride) was purchased from Tocris Bioscience (Ellisville, MO, USA), the A2AR antagonist MSX-3 (3,7-dihydro-8-[(1E)-2-(3-methoxy-phenyl)ethenyl]-7-methyl-3-[3-(phosphonooxy)propyl-1-(2-propynyl)-1H-purine-2,6-dionedisodiumsalt hydrate) and the D2R antagonist eticlopride were obtained from Sigma-Aldrich (StLouis, MO, USA). CGS21680 was dissolved in DMSO, and MSX-3 and eticlopride were dissolved in sterile saline with 0.1 N NaOH for final PH = 7.4. Doses of CGS21680, MSX-3 and eticlopride were established on previous behavioral studies with using microinjection procedures. These studies showed the efficacy of intra-NAc microinjection of A2AR agonist CGS21680 (0–10 ng/site) inhibited cocaine-induced reinstatement behavior [17, 24], and MSX-3 (0–20μg/site) enhanced cocaine-induced reinstatement behavior dose-dependently in rats [17]. Eticlopride is a dopamine D2R-selective antagonist (> 100,000 fold D2/D1) [29], it has been reported that intra-NAc shell microinjection of eticlopride(1–10 μg/site) dose-dependently blocked cocaine-primed reinstatement of cocaine-seeking, and bilateral injection of eticlopride (0.1–1.0 μg/site) in the BLA blockaded opiate-dependent and withdrawn rats [30, 31]. Moreover, the effects eticlopride (1.0–3.0 μg/site) in the NAc on propofol self-administration behavior has been tested in our previously published study [32].

### Surgeries

The intravenous catheters implantations were performed as previously described [33]. The rats were implanted with chronically indwelling intravenous catheters under sodium pentobarbital anesthesia, and the catheters were flushed daily with 0.2 ml saline-heparin solution to maintain the patency. In addition, the rats were treated with penicillin B through the catheter once per day during 7-day recovery period to prevent infection. The intra-NAc injections (A/P + 1.5 mm, M/L ± 2.0 mm, D/V—6.7 mm) were through bilaterally implanted guide cannulae (20-gauge, Small Parts Inc., USA) [8].

### Stereotaxic AAV Injection

pAAV-ZsGreen-shRNA-ADORA2A and pAAV-ZsGreen-shRNA-control were purchased from Neuron Biotech Co., Ltd (Shanghai, China). Intra-NAc microinjections of recombinant AAV–shA2AR or AAV–shCTRL (250 nl/side, 6 × 10^12^ particles/ml) were conducted under pentobarbital anesthesia (50 mg/kg i.p.) over 5 min. The needle was retained for at least 5 min after finishing the injection for complete drug diffusion. The accuracy of the microinjection in the NAc was determined under a fluorescence microscope (n = 10) (Nikon, Japan).

### Intra-NAc Microinjection Procedure

To assess the effects of the agents of on cue-induced reinstatement of propofol self-administration behavior, sucrose self-administration and locomotor activities, the rats were treated with vehicle, CGS21680 (2.5, 5.0 and 10.0 ng/site), MSX-3 (5.0, 10.0 and 20.0 μg/site) or eticlopride (0.75, 1.5 and 3.0 μg/site) 10 min before the test session. Microinjection in the NAc was delivered through a previously indwelling infusion cannula with a microinjection pump (MD-1001, Bioanalytical System Inc., West Lafayette, IN, USA) in a volume of 0.3 μl over 10 min.

### Self-administration Apparatus

The specifications of the apparatus were with custom-made operant boxes with a size of 30 cm × 30 cm × 30 cm, and the hemline was 5 cm from the floor. The rats were trained self-administration of propofol through jugular injection with a 5-ml syringe attached to a special pump with a speed of 1.2 ml/min. The rats would receive a dosage of 1.7 mg/kg propofol injection through the intravenous catheter after one active nose-poke as a reward. Still, no injection was given after inactive nose-poke. There was a 30-s time-out period after each active nose-poke, but no injection or reward would be given if nose-poke occurred during this period. Sucrose particles were used in the food-maintained training. The fixed ratio 1 (FR1) training schedule was applied for both propofol self-administration and food-maintained training. The locomotor activity was detected with a special motor monitoring device (Panlab, Barcelone Spain) for general locomotor activity evaluation versus the addictive property. All the experimental procedures were automatically recorded by a computer with MED Associates interface that runs self-programmed software written in Borland Delphi 6.0.

### Propofol Self-administration Training

The training procedure of establishing the propofol self-administration behavior model in rats was the same as described above, with a daily 3-h training session during the consecutive 14 days under a FR1 schedule. The sessions ended when 3-h or 100 propofol infusions were reached. The number of active nose-poke and propofol infusions increased as the training proceeded and reached a stable state after the successive 14-day training. Variability of less than 10% in the last 3 sessions was seen as successfully modeled in propofol self-administration behavior [34]. Those rats that did not reach the criteria were excluded from further study. Twenty-two rats were trained for establishing propofol self-administration behavior model after AAV-shA2AR or AAV-shCTRL intra-NAc injection, and 2 rats did not reach the criteria of the successful establishment of propofol self-administration model. Thus, the other 20 rats were randomized for further study. In addition, 115 rats were trained for propofol self-administration without AAV injection. Finally, 110 rats were included for further cue-induced propofol reinstatement behavior test after intra-NAc microinjection of vehicle, CGS21680, MSX-3 or eticlopride.

### Propofol Withdrawal Procedure and Cue-Induced Reinstatement Seeking

The propofol-dependent rats returned to individual cages after the self-administration behavior training and the rats were not available to propofol-related cues and stimulus during the 14-day abstinence period [13]. Subsequently, the rats were moved to the original self-administration training chambers and exposed to the previous behavior-training context and related cues such as the house light, a green LED light and pump noise. The difference from the propofol self-administration training is that no drug infusion was transmitted after each active nose-poke over the 3-h test session. The numbers of active and inactive nose-poke were also automatically recorded by computer.

### Specific Experiments

#### Experiment 1

Rats withdrawal from propofol self-administration for 14 days were randomly assigned to AAV-shA2AR group or AAV-shCTRL group (n = 10). Then the rats were tested cue-induced reinstatement of propofol self-administration behavior in the same context as previous self-administration training performed on day 15.

#### Experiment 2

To further determine the role of adenosine A2AR in the NAc in modulating cue-induced reinstatement of propofol self-administration, the rats that successfully established propofol self-administration behavior and withdrawal from propofol for 14 days were randomly assigned to one of the following groups: intra-NAc administration of vehicle; one dose of an agonist of A2AR CGS21680 (2.5, 5.0 and 10.0 ng/site) (n = 10); the antagonist of A2AR MSX-3 (5.0, 10.0 and 20.0 μg/site) (n = 10) 10 min before the cue-induced reinstatement of propofol self-administration behavior test.

#### Experiment 3

To evaluate the possible impact of dopamine D2R in the NAc on propofol relapse, the propofol-dependent rats with an abstinence period were randomly assigned to vehicle or eticlopride (0.75, 1.5 and 3.0 μg/site) (n = 10) 10 min before the cue-induced reinstatement of propofol self-administration behavior test.

### Sucrose Self-administration Training

Rats were trained self-administration for food reward with sucrose pellets (Dustless precision pellets, Bio-Serv, USA) under a FR1 schedule during a 0.5-h session for consecutive 7 days (n = 8). The paradigm was similar to that of propofol self-administration training, but the reward was replaced with a 45-mg sucrose for each active nose-poke. The sessions ended after either 0.5 h or 100 sugar pellets occurred. All rats reached the criteria of successfully modeled self-administration behavior for food reward with sucrose pellets.

### Locomotor Activity Test

The rats were tested to monitor the effects of intra-NAc injections of AAV vectors (AAV-shA2AR and AAV-shCTRL), CGS21680, MSX-3 and eticlopride on general activities with 1-h acclimation and followed by a 3-h test period (n = 6).

### Immunohistochemistry

The rats (n = 10) were fixed transcardiacally with 4% paraformaldehyde in 4 °C suspended in a phosphate-buffered saline solution (PBS, 0.1 M, PH = 7.4). Brains were removed and post-fixed in 30% sucrose until immersion at 4 °C and were sliced into 40-μm thickness. Immunohistochemistry was performed as described earlier [35]. In brief, sections were rinsed in PBS, incubated in 3% hydrogen peroxide in PBS for 30 min at room temperature (RT), and then incubated in 3% normal donkey serum, and 0.25% Triton X-100 in PBS (PBT) for 1 h sequentially at RT. After incubation in the primary antibody of mouse anti-A2AR (1:2000; Millipore, USA) at 4 °C overnight, the sections were rinsed and incubated in secondary antibody (biotinylated goat anti-mouse 1:500, Beyotime, China) for 2 h at RT. All slices were treated as introducing the ABC Elite kit (Vector, CA, US), and histological changes were visualized with a microscope (Nikon, Japan).

### Statistical Analysis

Continuous data were presented as mean ± standard deviation, and normality of data distribution was tested before further analyses. For the normal distributed data, the Student’s *t-*test was applied to two groups; and one-way analysis of variance (ANOVA) was used to compare multiple groups when the data also meet homogeneity of variance. Dunnett's post hoc test was adopted for multiple comparisons if any significant difference was found by ANOVA analysis. The data from repeated measurements were analyzed with repeated measures analysis of variance. Mann–Whitney U test and Kruskal–Wallis test were used for data were not normally distributed, and the Dunn’s post hoc analysis was used for multiple comparisons when a significant difference was found. Statistical calculations were performed with SPSS 25.0 (SPSS Inc, Chicago, USA), and a p-value smaller than 0.05 was considered significant.

## Results

### Deletion of A2A Receptor Expression in the NAc after AAV-shA2AR Injection But Did Not Alter the Process of Establishing Propofol Self-administration Behavior Under the FR1 Schedule

**Fig. 2:**
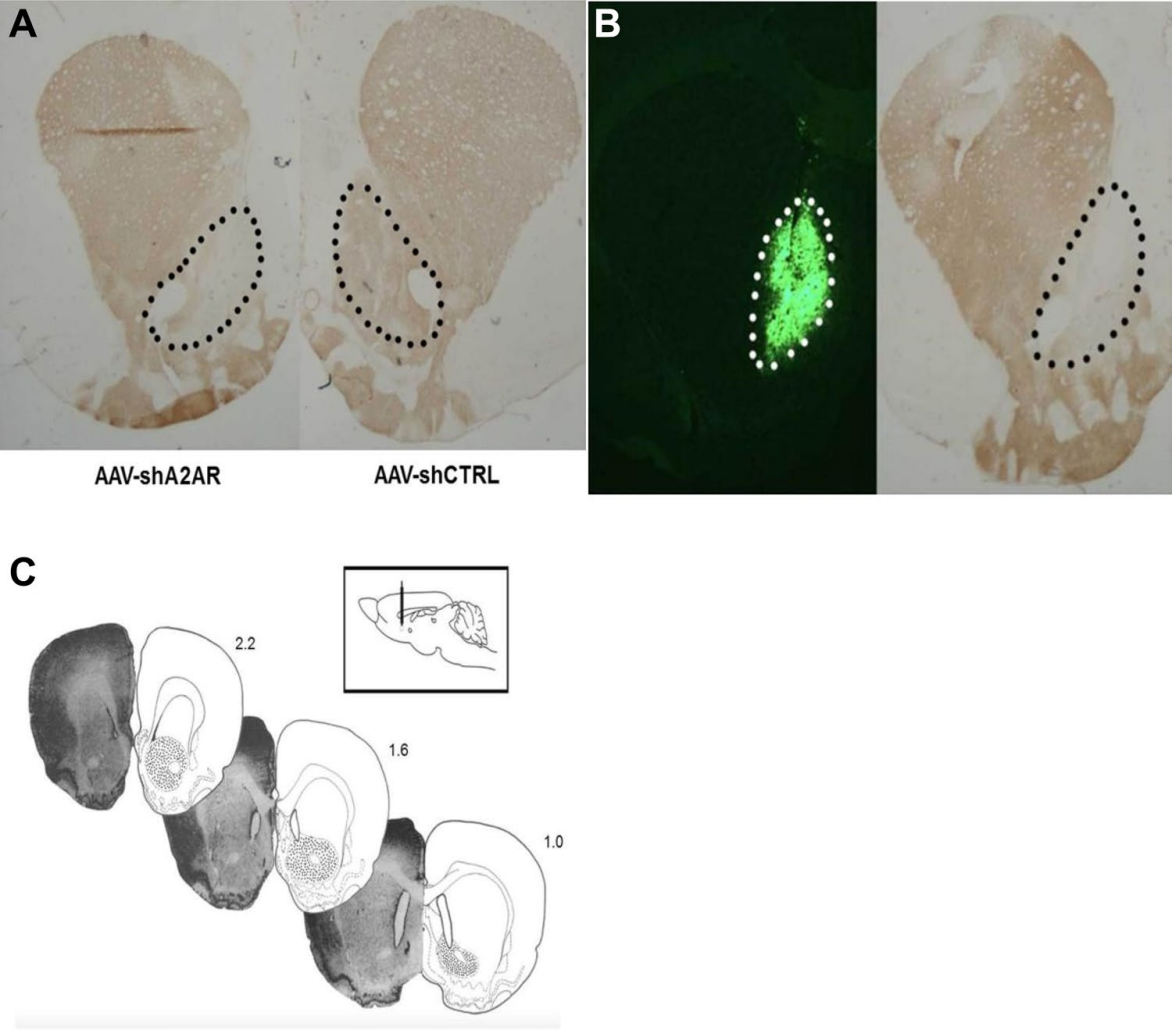
**a** Immunohistochemistry of the NAc slices that administration of AAV-shA2AR (left) or AAV-shCTRL (right), and the immunoreactivity for A2AR is significantly down-regulated in the NAc of the AAV-shA2AR treated rats compared with that treated with AAV-shCTRL. **b** AAV vectors that contained shA2AR are accurately injected in the NAc with green fluorescent (left), and the expression of A2AR is almost deleted after AAV-shA2AR injection (right). **c** Histological reconstructions of the rat brain to illustrate the microinjection in the NAc.

**Fig. 3:**
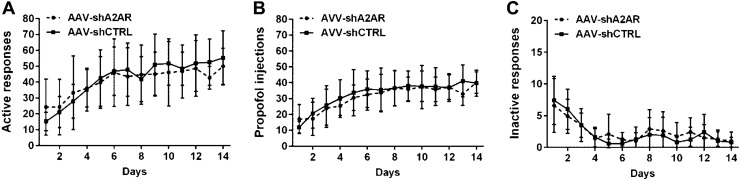
The numbers of active nose-poke response (**a**), propofol infusions (**b**) and inactive nose-poke response (**c**) were analyzed with repeated measures analysis of variance and show no difference in the group of AAV-shA2AR compared with the AAV-shCTRL group (p > 0.05, n = 10)

Figure 2 shows the expression of A2AR in the NAc was significantly reduced 3 weeks after stereotaxic injection of AAV-shA2AR compared with AAV-shCTRL group. The rats in the groups of AAV-shA2AR and AAV-shCTRL both developed reliable and stable propofol self-administration behavior within 14 days after the training, presenting an increase in active nose-poke response and propofol infusions and a decrease in inactive response. However, there were no significant differences between the two groups at either active nose-poke response (Fig. 3, F = 0.854, p = 0.602), infusions (F = 0.806, p = 0.570) or inactive nose-poke response (F = 0.589, p = 0.693), indicating that the establishing process of propofol self-administration under FR1 schedule was not mainly modulated by A2AR in the NAc.

### Deletion of Intra-NAc A2A Receptor Expression Facilitated the Cue-Induced Reinstatement of Propofol Self-administration Behavior in Rats

**Fig. 4:**
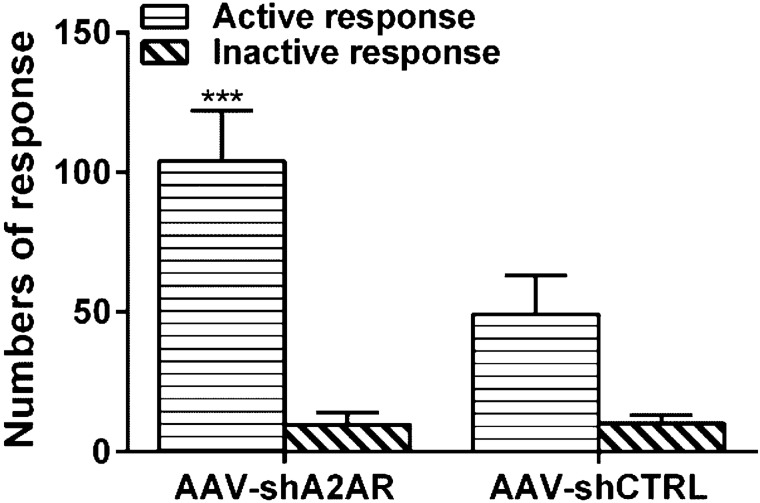
The cue-induced reinstatement of propofol self-administration behavior is facilitated by stereotaxic injection of AAV-shA2AR in the NAc after the 14-day abstinence period in propofol self-administrative rats. The numbers of active response increased significantly in the AAV-shA2AR group compared with AAV-shCTRL group (n = 10, p < 0.001), while no significant difference was found in the numbers of inactive response (p = 0.766). The data were analyzed with Student’s *t*-test, ***p < 0.001

The rats were trained to self-administer propofol 3 weeks after stereotaxic injection of AAV-shA2AR or AAV-shCTRL. The rats that experienced 14-day withdrawal were tested the cue-induced reinstatement of propofol self-administration behavior on day 15. The numbers of active nose-poke response were statistically enhanced compared to AAV-shCTRL group (Fig. 4, t = 7.614, p < 0.001), yet no significant evidence was found of inactive nose-poke response (t = -0.303, p = 0.766).

### Different Effects of Bilateral Intra-NAc Pretreatment with CGS21680 and MSX-3 on Cue-Induced Reinstatement of Propofol Self-administration Behavior

**Fig. 5:**
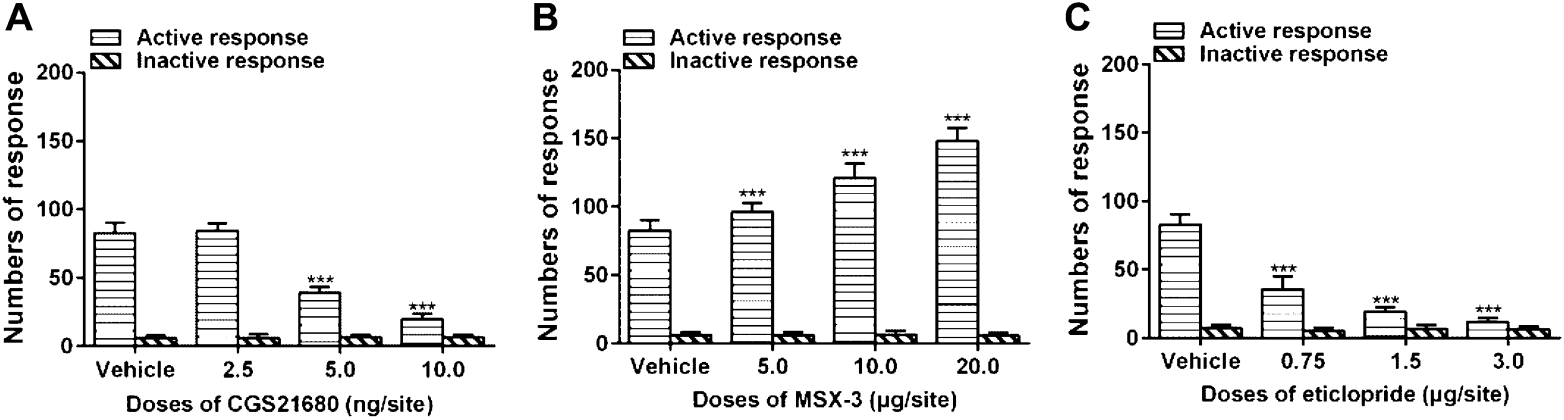
**a** Intra-NAc injection of an agonist of A2AR CGS21680 that pretreated 10 min before the cue-induced reinstatement of propofol self-administration behavior test attenuated active nose-poke response at the doses of 5.0 and 10.0 ng/site (n = 10, p < 0.001), but the numbers of inactive nose-poke did not reach a significance between the CGS21680 treated groups and the vehicle group (p = 0.928). **b** Intra-NAc pretreatment of the antagonist of A2AR MSX-3 stimulated the cue-induced reinstatement of propofol self-administration behavior, with the numbers of active nose-poke response significantly increased at the doses of 5.0–20.0 μg/site compared with the vehicle group (n = 10, p < 0.001), but the difference was not significant in inactive nose-poke response (p = 0.925). **c** Pretreated by the antagonist of D2R eticlopride in the NAc inhibited the cue-induced reinstatement of propofol self-administration behavior, with reducing the numbers of active nose-poke response (n = 10, p < 0.001), but did not affect the inactive nose-poke response (p = 0.414). The data analyzed with one-way ANOVA with Dunnett's pos hoc test for multiple comparisons, ***p < 0.001.

The rats trained to propofol self-administrated and withdraw for 14 days were pretreated with either vehicle or CGS21680 (2.5, 5.0 and 10.0 ng/site) by bilateral intra-NAc microinjections 10 min before the cue-induced reinstatement of propofol self-administration behavior testing session on day 15. It was found that the CGS21680 inhibited the numbers of active nose-poke response at the doses of ≥ 5.0 ng/site compared with the vehicle group (Fig. 5a, F = 339.355, p < 0.001; 5.0 ng/site p < 0.001, 10.0 ng/site p < 0.001), and the effect was not significant at the dose of 2.5 ng/site (p = 0.832). The numbers of inactive nose-poke response were not affected by any dose of CGS21680 (F = 0.151, p = 0.928).

The rats were pretreated with either vehicle or MSX-3 at the doses of 5.0, 10.0 and 20.0 μg/site 10 min before the relapse behavior test on day 15. The results indicated that MSX-3 dose-dependently increased the numbers of active nose-poke response compared with the vehicle group (Fig. 5b, F = 111.376, p < 0.001; 5.0 μg/site p = 0.003, 10.0 μg/site p < 0.001, 20.0 μg/site p < 0.001), but the difference of the numbers of inactive nose-poke response was not significant among the groups (F = 0.156, p = 0.925).

### Bilateral Intra-NAc Pretreated Eticlopride Inhibited the Cue-Induced Reinstatement of Propofol Self-administration Behavior

To explore the role of D2R in the NAc on cue-induced reinstatement of propofol self-administration behavior, the rats were bilaterally intra-NAc microinjected vehicle or one of the doses of eticlopride (0.75, 1.5 and 3.0 μg/site). The numbers of active nose-poke response were decreased by eticlopride compared with the vehicle group in a dose-dependent manner (Fig. 5c, F = 249.861, p < 0.001; 0.75 μg/site p < 0.001, 1.5 μg/site p < 0.001, 3.0 μg/site p < 0.001), but no significance was found of inactive nose-poke response (F = 0.977, p = 0.414).

### Microinjection of AAV-shA2AR, CGS21680, MSX-3 or Eticlopride in the NAc Did Not Alter the Sucrose Self-administration Behavior and Locomotor Activities.

**Fig. 6:**
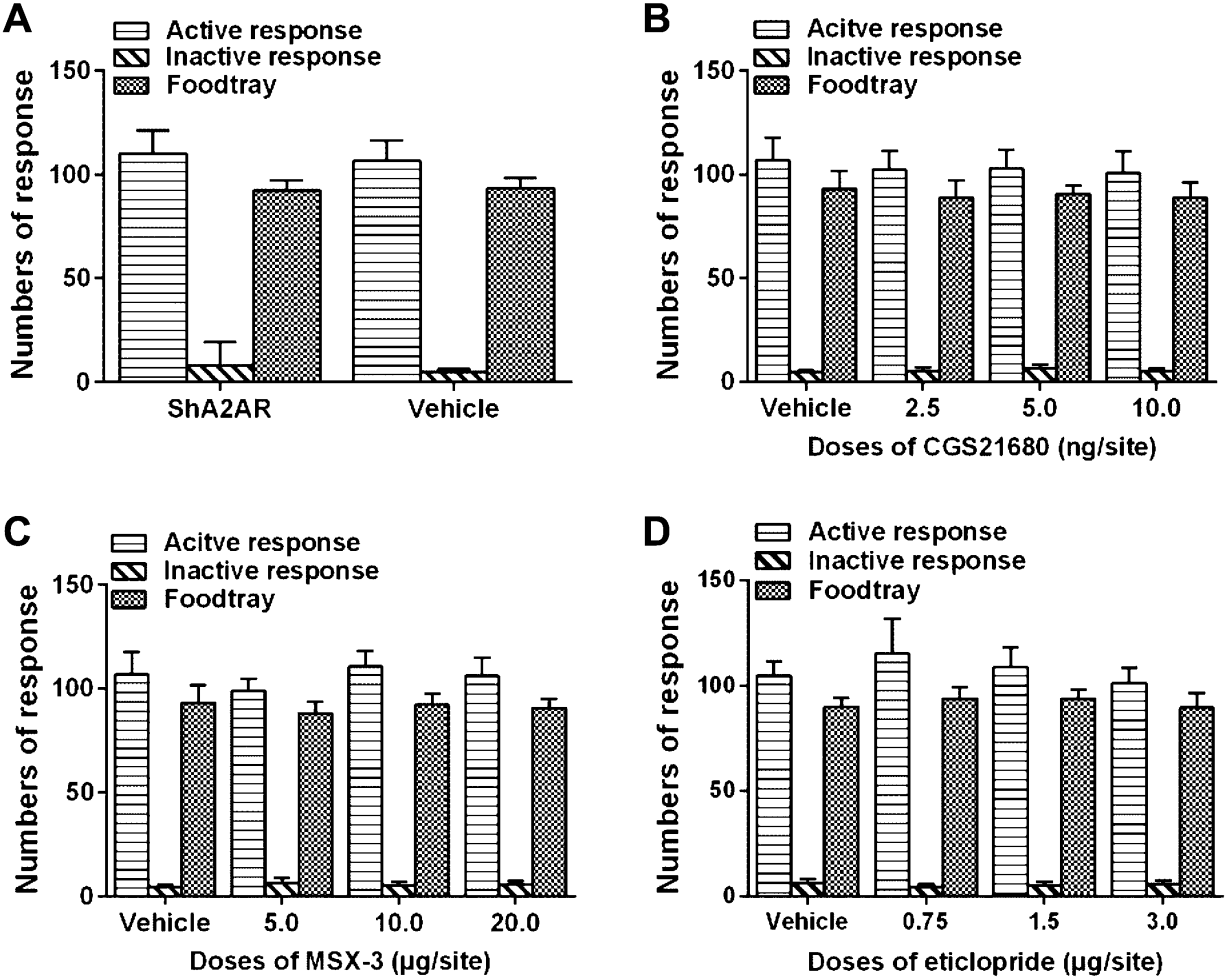
The stable sucrose self-administration behavior was established after a successive 7-day training period, and all the pretreatments of AAV, CGS21680, MSX-3 and eticlopride did not affect the sucrose self-administration behavior. One-way ANOVA analyzed the data of CGS21680, MSX-3 and eticlopride. Student’s *t*-test analyzed the numbers of active response and foodtray between AAV-shA2AR and AAV-CTRL (vehicle) groups. However, the numbers of inactive response were analyzed by the Mann–Whitney U test due to non-normal distribution (p > 0.05, n = 8)

**Fig. 7:**
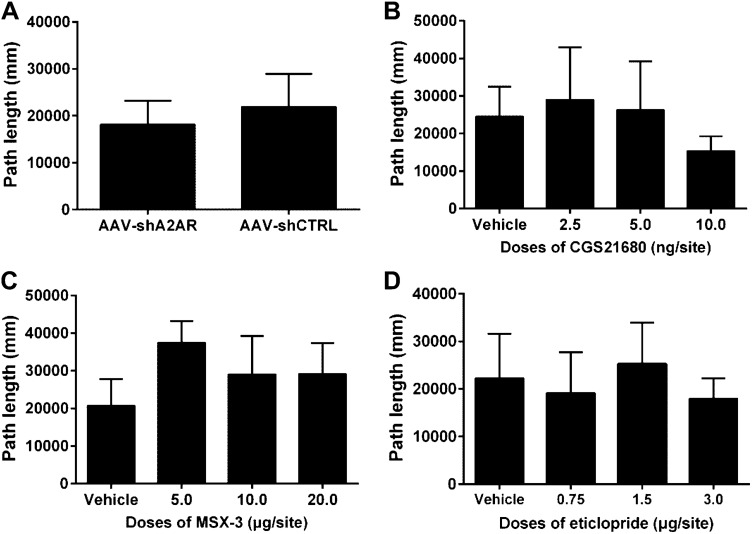
The effects of intra-NAc pretreatment of AAV (p = 0.192), CGS21680 (p = 0.160), MSX-3 (p = 0.135) and eticlopride (p = 0.408) on general locomotor activity were tested. The results show that all the treatments did not affect path length compared to the vehicle group (n = 6). Student’s *t*-test analyzed the data in AAV groups, and in eticlopride groups were by one-way ANOVA. Due to non-normal distribution, path length in CGS21680 and MSX-3 groups were analyzed by the Kruskal–Wallis test

To investigate the specificity of the pretreatments of AAV, CGS21680, MSX-3 and eticlopride, their effects on sucrose self-administration and general locomotor activities were examined. The sucrose self-administration behavior in rats was successfully established after a 7-day training, the numbers of active nose-poke response and sucrose pellets increased as training proceeded and stabilized at a high level, the numbers of inactive nose-poke response decreased and maintained at a minimal level, and the final test was performed on day 8. The stereotaxic injections of AAV and the other pretreatments of CGS21680, MSX-3 and eticlopride were completed as mentioned above before behavior tests. All these pretreatments failed to affect the numbers of active nose-poke response (Fig. 6, AAV t = 0.666 p = 0.516, CGS21680 F = 0.562 p = 0.644, MSX-3 F = 2.679 p = 0.066, eticlopride F = 2.469 p = 0.083), sucrose pellets (AAV t = -0.408 p = 0.689, CGS21680 F = 0.606 p = 0.616, MSX-3 F = 0.985 p = 0.414, and eticlopride F = 1.506 p = 0.235) or inactive nose-poke response (AAV p = 0.878, CGS21680 F = 1.883 p = 0.155, MSX-3 F = 1.045 p = 0.388, and eticlopride F = 2.318 p = 0.097). In addition, none of these pretreatments altered the locomotor activity of the rats as judged by path length (Fig. 7, AAV t = -1.357 p = 0.192, CGS21680 H = 5.95 p = 0.160, MSX-3 H = 5.55 p = 0.135, eticlopride F = 1.012 p = 0.408).

## Discussion

In our study, we found that the establishment of propofol self-administration behavior model was not affected by stereotaxic injection of AAV-shA2AR, but the cue-induced reinstatement of propofol self-administration behavior was significantly facilitated. Besides, our evidence shows that CGS21680 inhibited but MSX-3 promoted the cue-induced reinstatement of propofol self-administration behavior, and intra-NAc administrated with the antagonist of D2R eticlopride attenuated in the withdrawal rats. We speculated that A2AR mediated propofol relapse might be via D2R in the NAc. In addition, we also found that all treatments did not affect sucrose self-administration behavior or locomotor activities. Overall, these findings suggested that A2AR in the NAc plays critical role in mediating the cue-induced reinstatement of propofol self-administration behavior, which may act through D2R in the NAc with an antagonistic interaction.

Behaviors studies indicate that A2AR is linked to different stages of psychostimulant addiction, including drug abuse maintenance, withdrawal as well as reinstatement [36]. It has been determined that A2AR form structural heteropolymers with D2R with negative interaction, the latter also highly concentrate in the dorsal and ventral striatum [37, 38]. A2AR knockout mice exhibited increased ethanol preference and consumption but decreased morphine self-administration and conditioned place preference, and systemic CGS21680 treatment reduced cocaine self-administration infusions [23, 39, 40], suggesting that the neurobiological mechanisms in different drug addiction might be distinct. On the contrary, we found propofol self-administration behavior was not affected by down-regulated the expression of A2AR in NAc in this study. Our published study reported that propofol self-administration was mainly mediated by dopamine D1 receptor but not D2R in the NAc [32], different from the drugs of cocaine and morphine self-administration and CPP behaviors, which were mainly regulated by both D1 receptor and D2R [41, 42]. These findings may partially explain the different regulating effects of A2AR in the NAc on seeking behavior between propofol and other drugs.

It seems the underlying molecular mechanisms that contribute to different addiction stages of drug abuse are also distinct. Unlike the results of establishing propofol self-administration behavior, the cue-induced reinstatement of propofol self-administration behavior was significantly changed after genetically and pharmacologically regulating A2AR in the NAc. The results indicated a critical role of A2AR in the cue-induced reinstatement of propofol. Moreover, the cue-induced reinstatement of propofol self-administration behavior was also inhibited by D2R antagonist eticlopride. Our results are in line with previous research that morphine withdrawal were increased in A2A knockout mice [22], and single systematic administration of eticlopride attenuated cue-elicited nicotine reinstatement [43]. These findings also suggest that A2AR mediates propofol relapse probably by interacting with D2R signaling in the NAc.

A2AR were mostly distributed at postsynaptic of glutamatergic terminals and GABAergic striatopallidal projection neurons in the striatum, producing significant effects in altering dopaminergic signaling via several mechanisms, including direct receptor-receptor interaction, interaction at the second messenger level, trans-synaptic or at post-synaptic level [19]. A2AR interactions between other cell-surface receptors have been described, including dopamine D1 receptor, and subtype 5 metabotropic glutamates (mGlu5) receptors [19]. mGlu5 receptor mainly localized on postsynaptic elements that control higher cognitive and incentive function in the brain, the interactions between mGluR5 and NMDA receptors is vital for synaptic plasticity [44]. It has been demonstrated that the stimulation of mGluR5 enhanced NMDA receptor responses was mainly by phosphorylating NR2B subunit at the Tyr1472 site, and this reaction was enabled by A2AR [45]. Our studies have demonstrated that propofol addiction was regulated by ERK1/2 and c-Fos expression in the NAc [8, 18], and the signaling pathway NMDAR-D1R/ERK/CREB in the NAc was reported playing a critical role in control reward-seeking behavior [46]. Interestingly, it was also demonstrated that the co-stimulation of mGluR5 and NMDA receptor synergistically active ERK1/2 signaling and lead to c-Fos expression, and the reactions were under the permissive control of A2AR [44]. Therefore, based on the findings of previous research, we speculated that the modulation of A2AR in the NAc on cue-induced reinstatement of propofol self-administration might be via the signaling pathway of A2AR receptor/mGluR5/NR2B(Tyr1472)-D1 receptor/ERK1/2/c-Fos.

Adenosine A2AR in the brain also take part in modulating the general locomotor activity, but A2AR in different brain regions may not act uniformly. The striatal-specific knockdown A2AR enhances cocaine-induced locomotor activity, while the forebrain-specific knockdown of A2AR inhibits in response to cocaine [47]. Moreover, systemic pretreatment with CGS21680 reduces locomotor activity at high doses, but intra-NAc pretreated with CGS21680 at the doses from 0.5 to 10 ng per side did not produce any significant impact on cumulative cocaine-induced locomotor activity or sucrose reinstatement, which is consistent with the results in this study [17]. Taken together, these findings suggest that A2AR regulate locomotor activity response to psychoactive medications, but localizations of A2AR in the brain may affect differently.

Some other limitations of this study should be mentioned. As an addition to levodopa, the antagonist of A2AR Neurianz® (istradefylline) has been approved by US Food and Drug Administration (FDA) for Parkinson’s disease treatment after more than two decades of preclinical and clinical studies [48]. However, whether the antagonist of A2AR would aggravate drug abuse relapse, especially propofol relapse, remains an open question. Although the results indicated that the agonist of A2AR CGS21680 inhibited propofol relapse, based on the results of CGS21680 not only decreased the cocaine reinforcement behavior, the natural reward of food self-administration was also significantly inhibited [49]. Thus, we speculated that CGS21680 would produce a severe impact on general condition if was administrated systematically by oral? Moreover, despite the results indicated an essential role of the D2R in the NAc in propofol relapse, the expression of the D2R and down-stream underlying molecular mechanisms were not determined in this study. It is worth highlighting that the A2AR was also expressed in the brain regions of cerebral cortex, amygdala, olfactory bulb, but we just examined the A2AR in the NAc. Conditional A2AR knockout mice in different brain regions can be used and may provide further evidence of the effects of A2AR in different regions on the addictive property of propofol. All these questions need to be elucidated in the future.

The study provides clear evidence that deletion or antagonizes A2AR with MSX-3 in the NAc promoted cue-induced reinstatement of propofol self-administration behavior after withdrawal for 14 days while inhibiting propofol relapse by activating A2AR or antagonizing D2R. This study supports the assumption that A2AR in the NAc directly regulates propofol relapse by mediating the dopamine D2R in the NAc.

## Data Availability

The data used to support the findings in this study are available from the corresponding author upon reasonable request.

## Abbreviations

A2AR: A2A receptor
D2R: Dopamine D2 receptor
DA: Dopamine
NAc: Nucleus accumbens
BLA: Basolateral amygdala
FR1: Fixed ratio 1
CPP: Condition place preference
